# Supplementary material for: The Fetal Region-specific Optimized Growth Standard (FROGS)—A fetal and birthweight centile calculator validated in a national population
Source: PLoS Med. 2025 Jun 20;22(6):e1004634. doi: 10.1371/journal.pmed.1004634 (PMC12212869; doi:10.1371/journal.pmed.1004634)
Supplement: S1 File — (DOCX) [file pmed.1004634.s001.docx]

Design our own birthweight calculator

**Design our own adjustable sex-specific intrauterine growth centile calculator providing an exact centile for each gestational age.**

**PRE-SPECIFIED STATISTICAL ANALYSIS PLAN**

**Version 5.0, September 2023**

Table of Contents

[Research team 2](#_Toc101268475)

[Background 2](#_Toc101268476)

[Growth standard elements and rationale 2](#_Toc101268477)

[Technical aspects 3](#_Toc101268478)

[Translating to clinical practice 5](#_Toc101268479)

[Validating the calculator 5](#_Toc101268480)

[References 7](#_Toc101268481)

# Research team

**Dr Natasha Pritchard**

PhD Candidate, MBBS (Mercy Perinatal/ University of Melbourne)

**Dr Anthea Lindquist**

Perinatal Epidemiologist, FRANZCOG (Mercy Perinatal/University of Melbourne)

**Professor Stephen Tong**

Clinician scientist, FRANZCOG (Mercy Perinatal/University of Melbourne)

**Professor Susan Walker**

Maternal fetal medicine specialist, FRANZCOG (Mercy Perinatal/University of Melbourne)

**Dr Richard Hiscock**

Statistician and anaesthetist, ANZCA (Mercy Hospital for Women)

**Dr Emerson Keenan**

Research Fellow, PhD (Mercy Perinatal)

# Background

Obstetric growth standards are used to classify fetal and infant size. They are important, as classification of an infant as small or large has substantial implications for management throughout pregnancy (1, 2)**.** However, there are a multitude of different methodological approaches to deriving growth standards. The past decade has seen extensive debate over the merits and disadvantages of various methodlogies (3-5).

Most obstetric growth standards are derived from one of the following approaches:

1. Deriving growth standards from the averages of all infants born at a given gestational age within a given population (6, 7) (population charts)
2. Deriving growth standards from the averages of all infants growing in utero at a given gestational age within a given population (8, 9) (fetal charts)
3. Deriving growth standards only from the averages of healthy infants or fetuses (10, 11), often those who have only been exposed to optimal intrauterine environments (prescriptive charts)
4. Adjusting growth standards for maternal or fetal characteristics, and thus providing an individualized centile for a given fetus (12) (customised charts)

We aim to design a simple, easy to use, birthweight centile calculator incorporating the aspects with most value demonstrated within the existing literature and our prior research. We then aim to validate the calculator within a state-wide, population dataset, by assessing whether it better correlates with perinatal mortality and morbidity than existing growth standards.

# Our growth standard elements and rationale

1. **Derived from a fetal curve (Hadlock’s formula) (8):**

- The International Federation of Gynaecology and obstetrics (FIGO) endorses the use of fetal charts (13)
- A fetal curve better represents the entire obstetric population (both born and unborn) at any given gestation, than a birthweight chart (14). This is because a higher proportion of infants born preterm are growth restricted (15)
- A fetal curve makes the growth standard suitable for use in both a preterm and ultrasound (in utero) population. This is important, as antenatal management decisions based on size are made based on ultrasound measurements (1, 2)
- Hadlock’s formula is widely in use, and is used in the GROW algorithm (the most widely used customised growth standard) (12). This demonstrates acceptability of this formula to the obstetric community.

1. **Sex specific:**

- Our prior research has indicated that a sex specific growth standard improves the correlation of small infant size and adverse perinatal outcomes
- Ultrasound advances in recent years make fetal sex a variable that can be measured during pregnancy (16)
- Birthweight charts (derived from a born population) are always sex specific (6, 10). Thus, our growth standard would align with published birthweight charts.

1. **Provide an exact centile that is specific for any given gestation in days:**

- Our prior research has indicated that providing a day specific centile improves the distribution of small infants throughout the gestational week.
- Many newer growth standards use gestation in days (11, 17, 18) – this would align our growth standard with other contemporary growth standards.
- An exact centile, specific for gestational days, is easy to incorporate in a calculator, and has no apparent disadvantage.

1. **Based on Australian population means (but modifiable for any given population if it was to be adopted elsewhere):**

- WHO and INTERGROWTH-21^st^ have demonstrated that mean birthweights differ across populations, even when including only healthy individuals (11, 19)
- FIGO recommends a growth standard that classifies approximately 10% below the 10^th^ centile and 10% above the 90^th^ centile (13, 20). Their rationale is that the smallest 10% of any population contains enough growth pathology for obstetric intervention on the basis of size to be acceptable to the obstetric community.
- Basing a growth standard on population means will achieve the above recommendation.

1. **Based on Lancet’s “Global fetal and birthweight calculator” (9):**

- The “Global fetal and birthweight calculator” utilises Hadlocks’ formula, as well as the concept of backwards proportionality utilized in the well known GROW centiles (12). This is the concept that while Hadlock’s formula remains constant throughout gestation, it remains in proportion to the mean birthweight of a population at 40 weeks’ gestation (which can be modified).
- Unlike GROW centiles, the Lancet calculator is transparent in its formula and thus completely reproducible for all future clinicians and researchers (9).
- It is published in a highly reputable international journal, thus adding validity to our calculator if we have derived ours from it.
- A version of this is currently in use in many locations throughout Victoria, demonstrating acceptability to the local obstetric community.

# Technical aspects

*Below is the technical appendix taken from Lancet’s Global Fetal and Birthweight calculator****.***


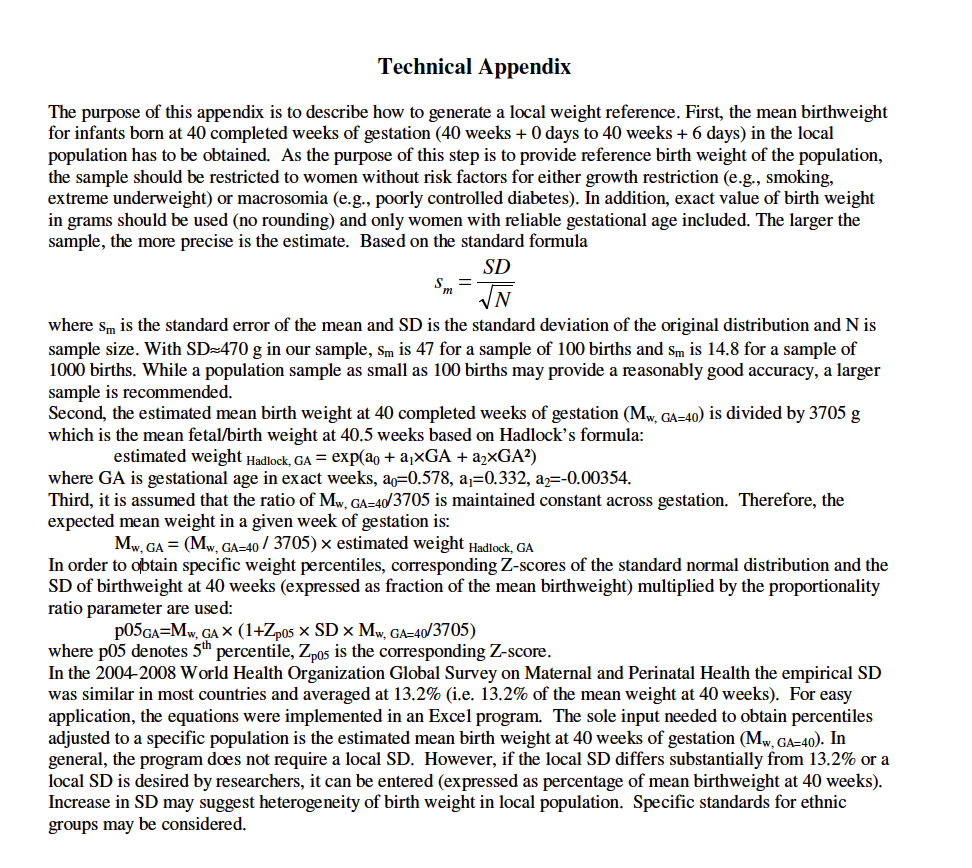


**Steps to derive the growth centiles:**

1. Identify the mean birthweight of infants born at 40 completed weeks’ gestation **(BW_40_)** (all infants born between 40+0-40+6 weeks). This mean birthweight can be either from the entire obstetric population, or from a healthy obstetric population only. Included pregnancies should be well dated. The average of the entirety of the week is used, as population birthweights are almost always available by week of getation.
2. Identify the standard deviation (SD) of the above mean birthweight at 40 weeks’ gestation and express it as a % of mean birthweight (in decimal format, eg. 11.5) **(SD_40_)**
3. Calculate the sex coefficient by dividing the mean difference between male and females within the population by 2.
   1. Create **BW_40M_ = BW_40_ + sex coefficient,** and **BW_40F_ = BW_40_ - sex coefficient**
   2. Use **BW_40_** as the default if no sex is specified
   3. If sex is specified, replace BW_40_ with the male or female version from this point onwards
4. Divide BW_40_ by 3705 grams (the mean birthweight at 40.5 weeks gestation by Hadlock’s formula).
   1. Hadlocks formula = exp(0.578 + 0.332x GA - 0.00354xGA^2^), where **GA** is the gestational age (in exact weeks)
5. Maintain the ratio of BW_40_/3705 across gestation, to obtain the mean birthweight for any given gestational age
   1. Mean BW_GA_ = BW_40_/3705 x estimated weight Hadlock_GA_
6. Generate birthweight percentiles across gestation by using Z-scores of the standard normal distribution, and the SD of birthweight at 40 weeks
   1. Eg. 5^th^ centile = Mean BW_GA_ x (1+Z_p05_ x SD x (BW_40_/3705))

In the case of our research, we plan to use mean birthweights from the 2019 AIHW dataset – the most contemporary published Australian birthweights:

- Mean birthweight at 40 weeks for male infants is 3641g, standard deviation 429g
- Mean birthweight at 40 weeks for female infants is 3504g, standard deviation 412g
- There were 392,131 male births and 386,154 female births at 40 weeks, with 778,285 total. Maintaining that ratio, the mean birthweight for *sex-unspecified* is 3573g

**Note regarding half/ full days:**

- There is a question of whether to input full days (eg. 168 days for 24.0 weeks) or half days (eg. 168.5 days for 24.0 weeks) into the calculator
- While full days may seem simpler, using half days aligns with the above technical appendix, and is more mathematically correct. The above formula, when referring to “40.5 weeks,” means 40 weeks +3.5 days.
- 0.5 days also aligns with using the mean birthweight for a full gestational week (ie. the mean birthweight for all births at 40 weeks’ gestation would be averaged upon 40.5 weeks)
- We have proceeded with the 0.5 days for accuracy

# Translating to clinical practice

For a simple, clinician friendly calculator, the only variables that would need to be inputted would be:

1. Actual birthweight or estimated fetal weight (grams)
2. Actual gestation of birth or ultrasound measurement (in days)
3. Sex – male, female or unknown

The aspects to the calculator that could be modified by researchers, but would not be routinely modified in a simple app would be:

1. Mean birthweight (can set this as a constant, so it is not modifiable by every clinician)
2. SD of mean birthweight within the population (can also set this as a constant)

With this information, an exact growth centile should be provided. We also propose to provide a confidence interval, based on a 10 or 15% margin of error (eg. the EFW is 10^th^ centile, range 5^th^ to 15^th^ centile).

# Validating the calculator

FIGO recommends two methods of validating a new growth standard (13):

1. Statistical validation: Finding the chart that best matches the distribution of weight within the local population, ie. a chart that follows a normal distribution, centered at the 50th percentile, and identifying approximately 10% of infants below the 10th percentile and 90% above the 90th percentile
2. Outcome based validation: Finding the chart for which the diagnosis of SGA is most predictive of adverse outcomes associated with fetal growth restriction.

We will aim to utilize both approaches, and can compare it to existing growth standards in use within Australia, to see if our proposed approach is beneficial.

We will compare our day and sex-specific calculator with a) the original Hadlock formula (8), b) the basic unadjusted weekly calculator provided by the Lancet paper (9) and c) population birthweight charts (which are currently used postnatally).

**Dataset:** Victorian Perinatal Data Collection data from 2009 – 2019 (approximately 850,000 total records). Data is routinely collected during pregnancy and birth.

**Inclusion criteria:** 24.0-42.6 weeks’ gestation, singleton, non-anomalous pregnancies.

**Exclusion criteria:**

- Congenital anomalies
- Multiple pregnancies
- Terminataion of pregnancy
- Missing or implausible data critical to determining a birthweight centile (sex, gestation in days, birthweight)

**Growth standard comparisons:** As a primary analysis, we will compare the growth standard described within this SAP to :

1. The original Hadlock estimated fetal weight formula. This is a growth standard derived from 392 middle class women from Texas in 1991, and provides an exact centile for each gestational day, but does not adjust for fetal sex or population means.
2. The ‘global reference for fetal-weight and birthweight percentiles’ proposed by Mikolajczyk et al. This uses the same formula as Hadlock above, and adjusts for population means, but provides only a single centile per completed week of gestation, and does not adjust for fetal sex.
3. Australian Institute of Health and Welfare chart. This is Australian population birthweight data, providing a single centile set per completed week of gestation. It is sex-specific.

**Part 1 - Statistial validation using birthweights:**

- We will plot the distribution of birthweights across all gestations using our custom chart, to assess whether the chart matches the distribution of weight within the local population.
- We will assess the distribution of birthweight centiles using a histogram for all relevant charts.
- We will describe proportions of infants classified as <10^th^ centile, <3^rd^, >90^th^ and >97^th^ centile by each chart
- Describe proportions of male and female infants classified as <10^th^ centile by each chart
- Describe proportions of infants classified as <10^th^ centile and <3^rd^ centile across the gestational week, ie. what proportion of SGA infants are born on the first day of the gestational week, the second day of the gestational week, etc.

**Part 2 – Outcome based validation:**

**Primary outcome:** Stillbirth.

**Secondary outcomes:**

1. Combined stillbirth and neonatal mortality
2. Apgars <4 at 5 minutes or <7 at 5 minutes
3. NICU admissions
4. Caesarean section rate (elective/ emergency/ total)
5. Induction or operative delivery indicated for poor fetal growth
6. Operative delivery for fetal distress

**Planned comparisons:**

*Primary analysis:*

Our primary comparison will be between our custom chart and each of the other charts listed above. We will compare these in the following ways:

- Compare the relative risk of stillbirth amongst <10^th^ centile infants by one chart, compared to the other
- Compare the relative risk of stillbirth and secondary outcomes amongst <10^th^ centile infants classified by one chart *but not the other* (ie. compare non-overlapping populations)

Outcomes will be presented using relative risks with 95% confidence intervals, and can be presented using attributable risk.

**Statistical analysis:**

- Analysis performed using Stata Version 16 (StataCorp. 2019. Stata Statistical Software: Release 16.1, College Station, TX, USA)
- Descriptive statistics will be constructed using mean and standard deviations for normally distribvuted data, median and interquartile ranges for skewed data, and number (%) if appropriate
- Statistical significance will be two sided, set at p<0.05 and not adjusted for multiple comparisons

**Adjustment for confounding factors:** This study is assessing the real life prognostic value of different obstetric growth standards. As such, we will not adjust for any potential confounders. This is because any differences between groups also reflect the classification process of the obstetric growth standard, and should not be adjusted for.

# References

1. Martins JG, Biggio JR, Abuhamad A. Society for Maternal-Fetal Medicine Consult Series #52: Diagnosis and management of fetal growth restriction: (Replaces Clinical Guideline Number 3, April 2012). Am J Obstet Gynecol. 2020;223(4):B2-b17.

2. Lees CC, Stampalija T, Baschat A, da Silva Costa F, Ferrazzi E, Figueras F, et al. ISUOG Practice Guidelines: diagnosis and management of small-for-gestational-age fetus and fetal growth restriction. Ultrasound in obstetrics & gynecology : the official journal of the International Society of Ultrasound in Obstetrics and Gynecology. 2020;56(2):298-312.

3. Gardosi J, Francis A, Turner S, Williams M. Customized growth charts: rationale, validation and clinical benefits. American Journal of Obstetrics and Gynecology. 2018;218(2, Supplement):S609-S18.

4. Papageorghiou AT, Kennedy SH, Salomon LJ, Altman DG, Ohuma EO, Stones W, et al. The INTERGROWTH-21(st) fetal growth standards: toward the global integration of pregnancy and pediatric care. Am J Obstet Gynecol. 2018;218(2s):S630-s40.

5. Selvaratnam RJ, Davey MA, Wallace EM. The pitfalls of using birthweight centile charts to audit care. PLoS One. 2020;15(6):e0235113.

6. Dobbins TA, Sullivan EA, Roberts CL, Simpson JM. Australian national birthweight percentiles by sex and gestational age, 1998-2007. The Medical journal of Australia. 2012;197(5):291-4.

7. Roberts CL, Lancaster PA. Australian national birthweight percentiles by gestational age. The Medical journal of Australia. 1999;170(3):114-8.

8. Hadlock FP, Harrist RB, Martinez-Poyer J. In utero analysis of fetal growth: a sonographic weight standard. Radiology. 1991;181(1):129-33.

9. Mikolajczyk RT, Zhang J, Betran AP, Souza JP, Mori R, Gülmezoglu AM, et al. A global reference for fetal-weight and birthweight percentiles. Lancet (London, England). 2011;377(9780):1855-61.

10. Villar J, Ismail LC, Victora CG, Ohuma EO, Bertino E, Altman DG, et al. International standards for newborn weight, length, and head circumference by gestational age and sex: the Newborn Cross-Sectional Study of the INTERGROWTH-21<sup>st</sup> Project. The Lancet.384(9946):857-68.

11. Kiserud T, Piaggio G, Carroli G, Widmer M, Carvalho J, Neerup Jensen L, et al. The World Health Organization Fetal Growth Charts: A Multinational Longitudinal Study of Ultrasound Biometric Measurements and Estimated Fetal Weight. PLOS Medicine. 2017;14(1):e1002220.

12. Gardosi J, Chang A, Kalyan B, Sahota D, Symonds EM. Customised antenatal growth charts. Lancet (London, England). 1992;339(8788):283-7.

13. Melamed N, Baschat A, Yinon Y, Athanasiadis A, Mecacci F, Figueras F, et al. FIGO (international Federation of Gynecology and obstetrics) initiative on fetal growth: best practice advice for screening, diagnosis, and management of fetal growth restriction. International journal of gynaecology and obstetrics: the official organ of the International Federation of Gynaecology and Obstetrics. 2021;152 Suppl 1(Suppl 1):3-57.

14. Ehrenkranz RA. Estimated fetal weights versus birth weights: should the reference intrauterine growth curves based on birth weights be retired? Archives of disease in childhood Fetal and neonatal edition. 2007;92(3):F161-F2.

15. Boulet SL, Alexander GR, Salihu HM, Kirby RS, Carlo WA. Fetal growth risk curves: defining levels of fetal growth restriction by neonatal death risk. Am J Obstet Gynecol. 2006;195(6):1571-7.

16. Kearin M, Pollard K, Garbett I. Accuracy of sonographic fetal gender determination: predictions made by sonographers during routine obstetric ultrasound scans. Australasian journal of ultrasound in medicine. 2014;17(3):125-30.

17. Papageorghiou AT, Ohuma EO, Altman DG, Todros T, Cheikh Ismail L, Lambert A, et al. International standards for fetal growth based on serial ultrasound measurements: the Fetal Growth Longitudinal Study of the INTERGROWTH-21st Project. Lancet (London, England). 2014;384(9946):869-79.

18. Nicolaides KH, Wright D, Syngelaki A, Wright A, Akolekar R. Fetal Medicine Foundation fetal and neonatal population weight charts. Ultrasound in obstetrics & gynecology : the official journal of the International Society of Ultrasound in Obstetrics and Gynecology. 2018;52(1):44-51.

19. Villar J, Papageorghiou AT, Pang R, Ohuma EO, Cheikh Ismail L, Barros FC, et al. The likeness of fetal growth and newborn size across non-isolated populations in the INTERGROWTH-21st Project: the Fetal Growth Longitudinal Study and Newborn Cross-Sectional Study. Lancet Diabetes Endocrinol. 2014;2(10):781-92.

20. Visser GHA, Nicholson WK, Barnea ER, Ramasauskaite D, Nassar AH, For the Figo Safe Motherhood NHC. FIGO position paper on reference charts for fetal growth and size at birth: Which one to use? International Journal of Gynecology & Obstetrics. 2021;152(2):148-51.
