# Supplementary material for: The Fetal Region-specific Optimized Growth Standard (FROGS)—A fetal and birthweight centile calculator validated in a national population
Source: PLoS Med. 2025 Jun 20;22(6):e1004634. doi: 10.1371/journal.pmed.1004634 (PMC12212869; doi:10.1371/journal.pmed.1004634)
Supplement: S1 Table — (DOCX) [file pmed.1004634.s003.docx]

**Supplementary Table 1: Baseline characteristics of cohort included in the analysis**

|  | **Overall cohort** |
| --- | --- |
| Birthweight (grams)  *Mean (SD)* | 3383 (542) |
| Gestational age at delivery (weeks)  *Median (IQR)* | 39.4 (38.6 – 40.3) |
| Proportion born in by gestational age (%)  <28 weeks  >=28 - <34 weeks  >=34 - <37 weeks  >=37 - <39 weeks  >=39 - <41 weeks  ≥41 weeks | 1,730 (0.23)  8,545 (1.13)  34,606 (4.58)  226,542 (30.0)  390,328 (51.7)  93,187 (12.3) |
| Maternal age (years)  *Mean (SD)* | 30.9 (5.3) |
| Male infant (%) | 385,162 (51.0) |
| Body Mass Index  *Median (IQR)* | 25 (22 – 29) |
| Nulliparous (%) | 331,333 (43.9) |
| Mother overseas born (%) | 270,798 (35.9) |
