# Supplementary material for: The Fetal Region-specific Optimized Growth Standard (FROGS)—A fetal and birthweight centile calculator validated in a national population
Source: PLoS Med. 2025 Jun 20;22(6):e1004634. doi: 10.1371/journal.pmed.1004634 (PMC12212869; doi:10.1371/journal.pmed.1004634)
Supplement: S2 Table — (DOCX) [file pmed.1004634.s004.docx]

**Supplementary Table 2: Proportion of infants born <3^rd^, <10^th^, >90^th^ and >97^th^ centile by each obstetric growth standard, by gestation**

|  | **FROGS** | **AIHW** | **Hadlock** | **Global** | **INTERGROWTH** |
| --- | --- | --- | --- | --- | --- |
| **TERM (n=651,103)** | | | | | |
| **<3^rd^ centile** | 19,242 (3.0%) | 17,135 (2.6%) | 15,696 (2.4%) | 20,851 (3.2%) | 5,670  (0.87%) |
| **<10^th^ centile** | 68,084 (10.5%) | 65,092 (10.0%) | 73,379 (11.3%) | 71,532 (11.0%) | 27,339  (4.2%) |
| **<50^th^ centile** | 346,058 (53.1%) | 354,187 (54.4%) | 421,510 (64.7%) | 348,005 (53.4%) | 237,239  (36.4%) |
| **>90^th^ centile** | 94,522 (14.5%) | 70,496 (10.8%) | 44,407 (6.8%) | 97,607 (15.0%) | 152,456  (23.4%) |
| **>97^th^ centile** | 39,896 (6.1%) | 22,004 (3.4%) | 14,125 (2.2%) | 42,135 (6.5%) | 67,855  (10.4%) |
| **>=34 AND <37 WEEKS’ GESTATION (n=34,606)** | | | | | |
| **<3^rd^ centile** | 3,709 (10.7%) | 1,006 (2.9%) | 3,264 (9.4%) | 3,735 (10.8%) | 1,554  (4.5%) |
| **<10^th^ centile** | 7,232 (20.9%) | 3,738 (10.8%) | 7,263 (21.0%) | 7,125 (20.6%) | 3,710  (10.7%) |
| **<50^th^ centile** | 19,608 (56.7%) | 17,529 (50.7%) | 22,284 (64.4%) | 19,193 (55.5%) | 14,771  (39.3%) |
| **>90^th^ centile** | 4,553 (13.2%) | 3,565 (10.3%) | 2,584 (7.5%) | 5,013 (14.5%) | 5,426  (15.7%) |
| **>97^th^ centile** | 2,334 (6.7%) | 1,162 (3.4%) | 1,188 (3.4%) | 2,618 (7.6%) | 2,247  (6.5%) |
| **>=28 and <34 WEEKS’ GESTATION (n=8,545)** | | | | | |
| **<3^rd^ centile** | 1,889 (22.1%) | 318 (3.7%) | 1,721 (20.1%) | 1,878 (22.0%) | 1,162 (13.6%) |
| **<10^th^ centile** | 2,835 (33.2%) | 971 (11.4%) | 2,842 (33.3%) | 2,846 (33.3%) | 1,808 (21.2%) |
| **<50^th^ centile** | 5,868 (68.7%) | 4,372 (51.2%) | 6,383 (74.7%) | 5,730 (67.1%) | 4,233 (49.5%) |
| **>90^th^ centile** | 637 (7.5%) | 845 (9.9%) | 369 (4.3%) | 760 (8.9%) | 1,083 (12.7%) |
| **>97^th^ centile** | 328 (3.8%) | 266 (3.1%) | 189 (2.2%) | 380 (4.4%) | 400 (4.7%) |
| **<28 WEEKS’ GESTATION (n=1,730)** | | | | | |
| **<3^rd^ centile** | 491 (28.4%) | 183 (10.6%) | 458 (26.5%) | 493 (28.5%) | 536 (31.0%) |
| **<10^th^ centile** | 638 (36.9%) | 318 (18.4%) | 640 (37.0%) | 666 (38.5%) | 650 (37.6%) |
| **<50^th^ centile** | 1,220 (70.5%) | 983 (56.8%) | 1,312 (75.8%) | 1,217 (70.3%) | 1,077 (62.3%) |
| **>90^th^ centile** | 106 (6.1%) | 148 (8.6%) | 65 (3.8%) | 116 (6.7%) | 198 (11.4%) |
| **>97^th^ centile** | 55 (3.2%) | 60 (3.5%) | 27 (1.6%) | 62 (3.6%) | 80 (4.6%) |
