# Supplementary figures and images for: The Fetal Region-specific Optimized Growth Standard (FROGS)—A fetal and birthweight centile calculator validated in a national population
Source: PLoS Med. 2025 Jun 20;22(6):e1004634. doi: 10.1371/journal.pmed.1004634 (PMC12212869; doi:10.1371/journal.pmed.1004634)

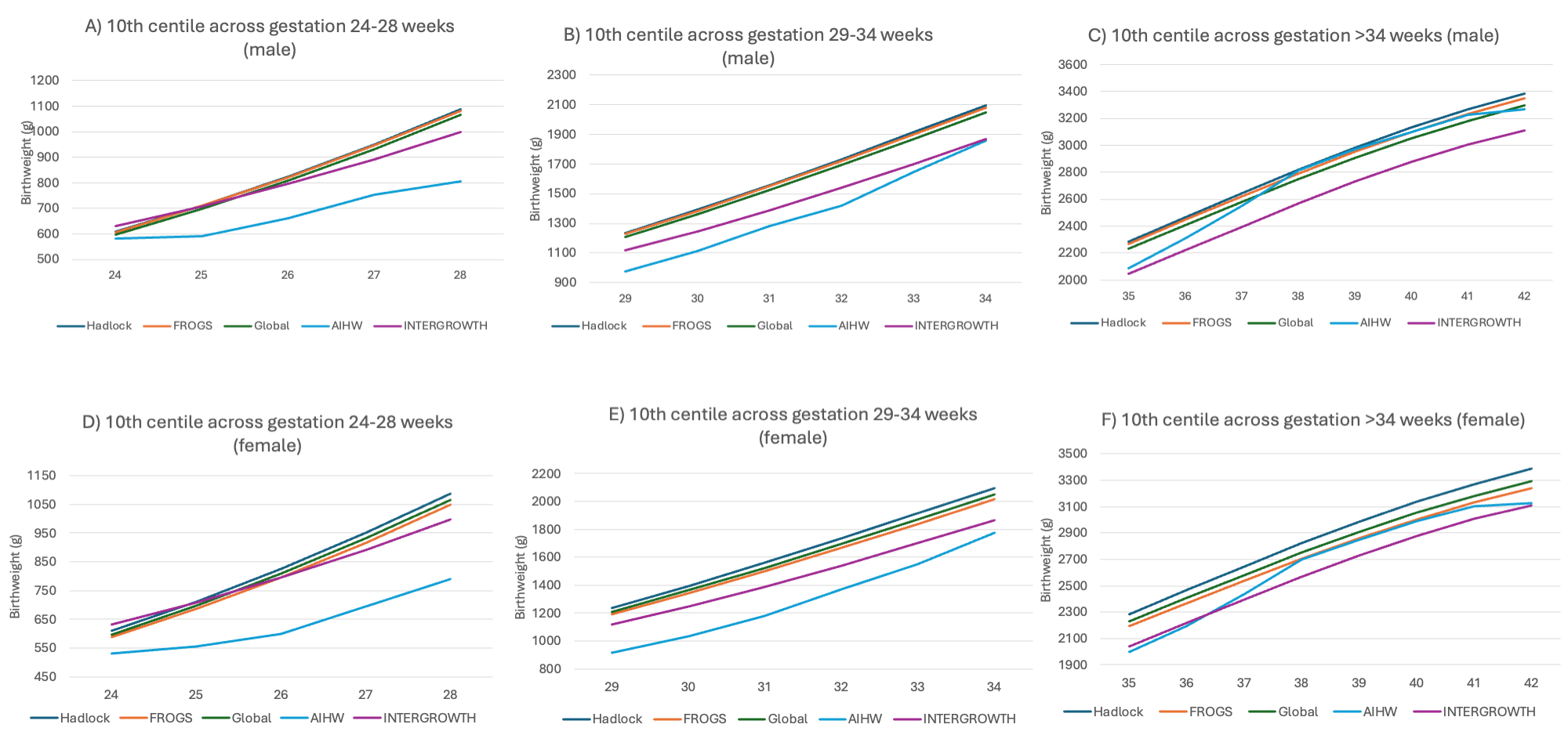

Supplement: S1 Fig — (TIFF) [file pmed.1004634.s005.tiff]
