# Supplementary material for: The Fetal Region-specific Optimized Growth Standard (FROGS)—A fetal and birthweight centile calculator validated in a national population
Source: PLoS Med. 2025 Jun 20;22(6):e1004634. doi: 10.1371/journal.pmed.1004634 (PMC12212869; doi:10.1371/journal.pmed.1004634)
Supplement: Attachment — Submitted filename: FROGS Rebuttal letter 21.4.25 V3.pdf [file pmed.1004634.s009.pdf]

REVISISIONS TO MANUSCRIPT PONE-D-22-14529

Dear Editors of PLOS Medicine,

We thank the reviewers and academic editors for their comments, and opportunity to submit our final revised manuscript. Please find our responses below.

Yours sincerely,

Dr Natasha Pritchard (corresponding author)

EDITOR COMMENTS:

1. **Abstract: 'Background' should be the rationale for the study, rather than the goal.**

Apologies for this error. We have changed the "Background" to read: "*There is no universally agreed upon obstetric growth standard for use during pregnancy. We aimed to design a simple novel growth standard, which incorporates key beneficial features identified in prior research."*

2. **Author summary: Last bullet point in 'why' should actually be in 'what'.**

Apologies for this. We have removed the last bullet point in the 'why' section and incorporated it as an abridged version in the 'what' section (as some information was duplicated.'

Legends: They're all there, but some don't provide enough information (eg. Don't mention what is in different panels), whereas others have a legend for each subpanel separately.

Apologies for this oversight. We have now updated the legends (in particular the supplementary figure legends) to reflect each subpanel separately.

3. **p18: You mention 'supplementary table 2s', please remove the 's'.**

Apologies for the oversight. This has now been corrected.

ACADEMIC EDITOR COMMENTS:

1) **Please compare FROGS to the fetal weight charts Intergrowth and WHO. As these are both used internationally and have online calculators, it would be good to provide a comparison to these fetal charts. If this is not possible, please provide some explanation why this has not been done.**

Thank you for these valuable comments. We agree that there would be more international applicability through comparisons with WHO and INTERGROWTH fetal charts. We are pleased to now incorporate a comparison of FROGS with the INTERGROWTH fetal weight chart. However, we have not included a comparison with the WHO chart. To our knowledge, there

is no easily accessible bulk online calculator for WHO, and it was not feasible to calculate the individual percentile for each infant given the size of our dataset.

We have included the results on statistical validation for all infants classified as SGA by INTERGROWTH, compared with those classified as SGA by FROGS.

All findings and methodological changes are incorporated and highlighted throughout the manuscript, and a summary of the key findings are below:

1. **STATISTICAL VALIDATION**
   - INTERGROWTH classified only 4.4% of the population below the 10th centile, but 21.1% above the 90th centile.
   - It has a significant right skew when applied to our population (as can be seen in the histogram below), with only 34.1% of infants falling below the 50th centile, and over one in five infants being classified as >90th centile.
   - Perhaps most notably, only 4.4% of infants fall below the 10th centile. This is compared to FROGS in which 3.4% of infants were classified as <3rd centile. The risk of stillbirth in infants classified as <10th centile by INTERGROWTH was 20.3 per 1000, while the risk of stillbirth in infants classified as <3rd centile by FROGS was 24.2 per 1000. This means that cohort of infants classified as small for gestational age by INTERGROWTH was, in size and risk, closer to the cohort classified as <3rd centile by FROGS (and other charts). The underestimation of SGA, and overestimation of LGA, limits clinical applicability of INTERGROWTH in our population.
   - As seen with the other non sex-specific comparison charts, INTERGROWTH classifies far fewer male infants (only 37.5%) as SGA.

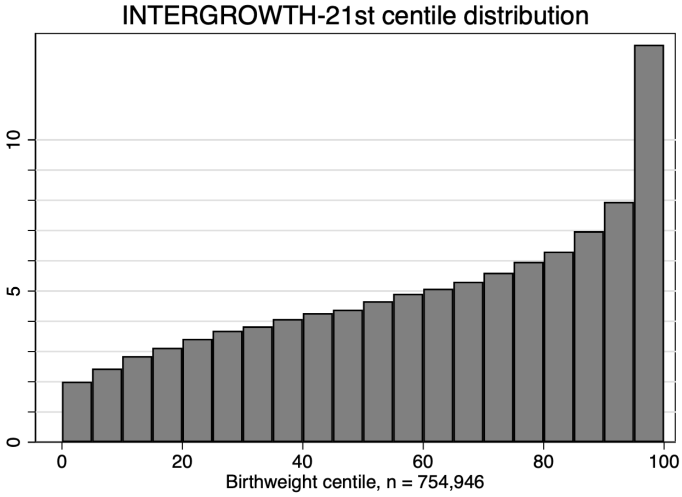

2. **OUTCOME BASED VALIDATION**
   This failure of statistical validation undermines meaningful outcome-based validation. The smaller proportion of infants classified as <10th centile by INTERGROWTH means that only 54 infants (0.007% of the total population) were classified as SGA by INTERGROWTH but not FROGS. This contrasts sharply with our other analyses, and means this non-overlapping population is not a robust comparator in an outcome-

based validation of the INTERGROWTH charts, given the lack of power and precision with such small numbers. We observed this in a past paper published in PLOS Medicine, in which we also compared INTERGROWTH with other existing growth charts among a preterm population, and we approached it similarly. (https://journals.plos.org/plosmedicine/article?id=10.1371/journal.pmed.1002923) .

2) **The discussion doesn't really tease out the thorny issue that adjusting for population means in a diverse population doesn't account for different rates of pathologically small babies in the different subgroup populations. I accept that FROGS is being presented as a middle ground but I think the point that adjusting for a local population where one group will much more commonly be <10th centile than another group still leaves the issue that you overtreat one group whilst undertreating another. FROGS gets you nearer to your local population but doesn't actually address this challenge at all. Clearly further research of adverse outcomes in subgroups of a population (eg. For our local population it would be a subgroup adjustment amongst our south Asian women) is needed to determine whether a further adjustment for the local population should be applied or not.**

Thank you for these comments. We agree that there are certain subpopulations that have an increased risk of adverse perinatal outcomes, who may also have different rates of small for gestational age infants. As raised by the academic editor, infants born to South Asian mothers may have increased rates of term stillbirth, as well as increased likelihood of being classified as small for gestational age. We acknowledge not all sub-populations behave similarly and there is inevitable local, and systemic, variation in stillbirth and SGA rates in sub-populations. There is no perfect solution. But given the complexity of social determinants of stillbirth risk in a multi-ethnic population, we suggest the most practical path forwards is not to adjust the weight centile for these factors, but rather to keep the weight centile static and overlay these risk factors in clinical decision-making. For example, while it is possible to adjust a fetal weight centile for parity, it is also possible to keep the fetal weight centile the same, and instead alter obstetric management decisions acknowledging that parity affects the risk of adverse outcomes. We have chosen a population-based approach, as previous research has suggested that there are population-based birthweight differences not fully explainable by maternal ethnicity, which may instead represent the multitude of social and environmental factors that can influence weight *(Kiserud et al. – The World Health Organization fetal growth charts: concept, findings, interpretation, and application).*

We have added a comment the discussion to further elucidate the issues at hand, *"On a population level, we propose FROGS as a simple, transparent, stable weight centile. This centile provides a robust baseline for clinical management decisions based on fetal size, on which other local, and systemic, risk factors for adverse perinatal outcomes (for example, ethnicity) can be laid. The application, and impact, of such adjustments should be a focus of future research"*

<u>REVIEWER #1 COMMENTS:</u>

1. **In this revised version of their manuscript they have addressed previous reviewer comments.**

Thank you for these comments and re-review.

__REVIEWER #2 COMMENTS:__

1. **Thank you for the opportunity to review this revised version of the article. I am pleased with the comprehensive responses the authors provided to my questions and the alterations that were made to the manuscript. If the article is accepted for publication, I hope the authors opt for publication of the peer review history because it provides a deepened and nuanced understanding of the article.**

Thank you for these comments. We also agree that the discourse relating to our manuscript in the previous revisions is valuable, and will opt for publication of the peer review history.

2. **I have one minor comment regarding the discussion section: the authors mention a "strength" in the second on research implications, this should be put in the strengths and limitations section.**

Thank you for identifying this. We have moved this to the strengths and limitations section. We have also moved these comments to the strengths and limitations section.

__REVIEWER #3 COMMENTS:__

1. **This paper has been revised and my comments have been largely addressed. As such it appears acceptable for publication.**

Thank you for the re-review.

__TECHNICAL REQUESTS:__

1. **Please ensure that the funders and grant numbers match between the Financial Disclosure field and the Funding Information tab in your submission form. Note that the funders must be provided in the same order in both places as well. Currently, "the NHMRC EL1 Fellowship" is missing form the Funding Information.**

Apologies for this oversight. All funders and awards are now in the identical order in both sections.

2. **Please amend your detailed Financial Disclosure statement. This is published with the article, therefore should be completed in full sentences and contain the exact wording you wish to be published. I) State what role the funders took in the study. If the funders had no role in your study, please state: "The funders had no role in study design, data collection and analysis, decision to publish, or preparation of the manuscript." II) If any authors received a salary from any of your funders, please state which authors and which funders.**

Thank you for this clarification. The funding details section now reads *"NP is the recipient of a Norman Beischer Innovation Grant (https://nbmrf.org.au/grants/) and an Avant Doctors in Training Postgraduate Scholarship (https://avant.org.au/foundation/member-grants). TM is the recipient of an NHMRC EL1 Fellowship, which provides salary support, and a Norman Beischer Medical Research Foundation Clinical Research Fellowship (https://nbmrf.org.au/grants/). The funders had no role in study design, data collection and analysis, decision to publish, or preparation of the manuscript."*

3. **The file inventory includes files for [Figures 2a, 2b, 3a, 3b, 4a to 4c]. We would recommend either combining these into a Figure 2,3,4 with separate internal panels, or renumbering them as individual figures, as we are not able to publish multiple components of a single figure as separate files.**

Thank you for this clarification. We have combined figures 2, 3 and4 into figures with separate panels.

4. **We notice that your supplementary Figure, tables are included in the manuscript file. Please remove them and upload them with the file type "Supporting Information." Please ensure that each Supporting Information file has a legend listed in the manuscript after the references list.**

Thank you for this clarification. Our supplementary files and tables are now individually uploaded and removed from the manuscript, with a legend after the references.

5. **We notice that your Supplementary Figures are duplicated on your submission as they are included in the manuscript file and are also uploaded separately as "Supporting Information" file. Please remove them and upload them with the file type "Supporting Information" and ensure that each Supporting Information file has a legend listed in the manuscript after the references list.**

Apologies, we have removed the duplicates within the manuscript.

6. **We have noticed that you have uploaded Supporting Information files, but you have not included a list of legends. Please add a full list of legends for your Supporting Information files after the references list.**

Apologies, we have now uploaded a list of legends.

7. **Please remove the 'conclusions' subheading.**

Apologies for this oversight in formatting. This has been removed.

8. **Please report statistical information as follows to improve clarity for the reader "22% (05%CI [13%, 28%\:p</=)". Please repeat statistical definitions (CI etc.) for each set of parentheses.**

Thank you, we have presented the statistical information in the above format.

9. **In the last sentence of the Abstract Methods and Findings section, please describe the main limitation(s) of the study's methodology.**

Thank you for raising this point. We have now added to the abstract *"This study is limited in that it retrospectively assesses birthweight, which may have different implications to a prospective evaluation of estimated fetal weight."*

10. **In the author summary, in the final bullet point of "What Do These Findings Mean?", please include the main limitations of the study in non-technical language.**

Thank you for raising this. We have now added "*The findings of this study might differ, if FROGS was applied to estimated fetal weights measured on an ultrasound in pregnancy, rather than to known birthweights after delivery"* to the section "What Do These Findings Mean?"

11. **The funding statement should include: specific grant numbers, initials of authors who received each award, URLs to sponsors' websites. Also, please state whether any sponsors or funders (other than the named authors) planned any role in study design, data collection and analysis, the decision to publish, or preparation of the manuscript. If they had no role in the research, include this sentence: "The funders had not role in the study design, data collection and analysis, decision to publish, or preparation of the manuscript."**

As above in response to point 2, this has now been done.

12. **Please include the statement on code availability in the data availability statement.**

Thank you for this. We have now included the statement "The author-generated code is available."

13. **Please show graph axes beginning at zero. If this is not possible, please show a break in the axis.**

Apologies for this. We have now shown a break in the axis in the instances where the graphs do not begin at 0.
