# Supplementary material for: The Fetal Region-specific Optimized Growth Standard (FROGS)—A fetal and birthweight centile calculator validated in a national population
Source: PLoS Med. 2025 Jun 20;22(6):e1004634. doi: 10.1371/journal.pmed.1004634 (PMC12212869; doi:10.1371/journal.pmed.1004634)
Supplement: Attachment — Submitted filename: FINAL FROGS Rebuttal letter 18.2.25.docx [file pmed.1004634.s008.docx]

**REVISISIONS TO MANUSCRIPT PONE-D-22-14529**

Dear Editors of PLOS Medicine,

We thank the reviewers for their considered comments and for the opportunity to provide a revised and improved manuscript. Please find our responses below.

We look forward to further correspondence regarding our manuscript.

Yours sincerely,

Dr Natasha Pritchard (corresponding author)

**Requests from the editors:**

1) **Reviewers #3 and #4 were concerned about some of the language used, and whether this adequately reflected your results. Reviewer #2 asked for comparisons of FROGS with additional birthweight charts, a point which the Academic Editor thought was important. After discussing the paper with the editorial team and an academic editor with relevant expertise, I'm pleased to invite you to revise the paper in response to the reviewers' comments. We plan to send the revised paper to some or all of the original reviewers, and we cannot provide any guarantees at this stage regarding publication.**

Thank you for these comments, and for the opportunity to revise our manuscript. We have addressed these comments below in response to the individual reviewer’s comments.

**REVIEWER # 1 COMMENTS:**

**This publication describes the comparison of four different charts for calculating birthweight centiles:  a novel, customised fetal Hadlock chart which the authors have named FROGS (Hadlock adjusted for local mean term birthweight and newborn sex); a standard, un-customised fetal Hadlock chart; a national Australian birthweight AIHW chart; and a published Mikolazcyk chart.**

**Overall, the manuscript is clearly written and well-presented and the methods are adequately described. This study is interesting and deserves wide dissemination. The authors argue successfully in favour of the FROGS chart, demonstrating that - although all four charts have similar screen positive rate for SGA at around 10% - the FROGS chart can capture more effectively pregnancies resulting in stillbirth (primary outcome) and other secondary outcomes. The authors conclude that the reasons why the FROGS chart performs better compared to the other three charts are:**

1) **Calculation of exact birthweight centile for each gestational day, as opposed to centile for completed week**
2) **Adjustment of the curve for the local population mean term birthweight and standard deviation.**
3) **Adjustment of the curve for the infant sex.**

**In my opinion, conclusion 1 is not a particularly novel or newsworthy. Since the early 1990s, it is universally accepted that gestational age should always be expressed in exact days when calculating reference ranges and percentiles, rather than using truncated weeks. Nevertheless, the comparisons presented here in the manuscript, quantify this effect neatly and eloquently.**

Thank you for your summary and comments. We strongly agree that gestational age should be consistently expressed in exact days rather than truncated weeks, although we note this is inconsistently applied in clinical practice. We hope that by highlighting the clinical benefits of using exact days, it will help standardise best practice .

**Conclusions 2 and 3 are interesting. The authors make a compelling argument that adjustment for local population mean birthweight and also infant sex customisation, will increase the capture of stillbirth, without significantly changing the screen positive rate.**

Thank you for this positive feedback.

1) **The authors present their methods transparently. However, both in the abstract and the discussion the authors make frequently a statement of opinion that "the FROGS centile development algorithm is simple, transparent and does not change with time. This means it can be easily adapted for local populations…" Although it is transparent, it is not simple to adapt prospectively in the delivery of prenatal care. It is also incorrect to state that it does not change with time. The fact that FROGS is adjusted for local birthweight means that it would need to change with time as mean birthweights change, either in different times or locations. The authors contradict their previous assertion in page 22 where they state "it can be updated easily if local mean birthweights change over time".**

Thank you for highlighting this valid point and allowing us to clarify. We agree with the reviewer that prospective adoption of any new growth standard is not necessarily easy, and we have removed those statements that are not supported by the data within our study (Abstract Conclusion page 3, Discussion page 21). We have also removed the phrase “does not change with time,” as we agree that the meaning was not clear. What we wished to highlight was that *the algorithm itself would be transparent and stable* (as opposed to being updated with new coefficients, which is the case with other existing customisation charts in use) (Discussion, page 26).

*“While the FROGS algorithm is transparent and stable, the derived centiles can be updated if local mean birthweights change over time.”*

2) **For a chart to improve prenatal care it should be used when reporting prenatal ultrasound and EFW centiles. Most institutions use a commercially available software (Viewpoint, Astraia etc) when reporting ultrasound examinations, so that the scan measurements are plotted in centile charts. The authors propose that every hospital or health board should be able to simply calculate their local population birthweight distributions after applying all sensible exclusions. Then, that they can modify the commercially available software. Or that they could design a bespoke electronic calculator from scratch. This is not really plausible. This is why the 1991 Hadlock chart remains in common use in 2024.**

Thank you for these comments, which accurately highlight some of the potential issues with implementation of a new obstetric growth standard compared to existing standards embedded into clinical software. While we agree that implementation is always challenging, publication of these findings would mean the online calculator (embedded in the manuscript) is universally available for research and database use- and potentially incorporated into local ultrasound software. At an individual level, we propose to convert the algorithm into a clinician co-designed app for widespread dissemination. This will enable individual clinicians to input the estimated fetal weight from the ultrasound into an app to obtain the relevant centile. We do believe that population-based birthweight data, which is all that is required to use our algorithm is one of the more easily and widely accessible epidemiological data points (Discussion, page 26).

*“While implementation of any clinical change always has pragmatic challenges, we believe that the ability of FROGS to generate a growth centile chart based on population-based birthweight data, one of the most widely accessible epidemiological data points, will limit the barriers to change.”*

3) **The authors make a compelling argument that customisation by fetal sex will improve the identification of SGA babies at risk of stillbirth. It is easy to do this when the baby is born where the sex is obvious. But the fetal sex is not universally recorded on prenatal ultrasound. This is not a mandatory part of prenatal care. In some parts of the world reporting the fetal sex prenatally is prohibited.**

Thank you for highlighting this very valid point. We feel that demonstrating the benefit of adjusting for fetal sex in identifying infants at greatest risk is important, but acknowledge that this may not be feasible or acceptable in some settings. Our calculator has provided the option of a sex-unadjusted growth centile value, for situations in which fetal sex is unknown, or unable to be determined. We also believe that there is the potential for sex to be noted by the sonographer but not disclosed to the parents; this happens frequently in clinical practice. A similar situation can be seen following non-invasive prenatal testing, where the results are not always disclosed to the patient. We have now highlighted this in our manuscript – Clinical Implications, page 26.

*“Our findings suggest benefit in adjusting for fetal sex. However, fetal sex is not always known antenatally, and in some countries reporting the fetal sex prenatally is prohibited, to avoid sex-selective termination of pregnancy (69). Using the sex-unadjusted option of the FROGS algorithm is a practical option in cases where sex is unknown, but may reduce the potential benefits of the FROGS chart. There is also the possibility for fetal sex to be noted by the sonographer, but not disclosed to the parents. A similar practice can be seen in the context of non-invasive prenatal testing, where results can be known to the clinician but not disclosed to the patient.”*

4) **In my opinion, customising EFW reports for sex and adjusting for reference charts for local birthweight distributions is scientifically interesting but not easy or practical. The authors make repeated statements to describe this as a strength, but instead they should have addressed this as a limitation.**

Thank you for raising this point about the challenges of implementation. We agree that making any practice change can be challenging. However, within our country, there is a wide variety of growth standards currently in use. Therefore, there is the potential for streamlined change. We have removed the statements suggesting that it is easy to translate, as per response to Reviewer 1 point 3.

**Specific comments:**

5) **Page 3 The statement "The FROGS centile development algorithm is simple, transparent and does not change with time. This means it can be easily adapted for local populations and applied to clinical and research settings globally.” This is a statement of opinion that is not supported by the facts presented here.**

Thank you for highlighting this potentially confusing statement. As discussed above, we have amended this sentence in the abstract to remove references to the fact that it does not change with time (Abstract, page 3). Instead, we now conclude *“The FROGS centile algorithm is simple and transparent. It has the potential to be adapted to other local populations or applied to clinical and research settings globally.”*

6) **Page 8 "The standard deviation at 40 weeks' gestation for both sexes combined was 421g, which, as a percentage of birthweight, was 11.8%. This was used in all cases,…"Did the authors use a fixed standard deviation of 421g across every gestational week from 20 to 42 weeks; or did they use a standard deviation calculated at each gestational age as 11.8% of the mean birthweight for that gestational age?**

Thank you for highlighting the ambiguity of this terminology. We did not mean that the absolute standard deviation of 421g was constant in all cases, but rather the standard deviation expressed as a percentage of birthweight (11.8%) was constant across gestational age. We have clarified this in the text (Methods, page 9).

*“For all earlier gestations, the standard deviation used to derive percentiles remained constant at 11.8% of the mean birthweight for any given gestational age.”*

7) **Page 10 Please define what was an implausible birthweight.**

Apologies for this omission in our methods. This dataset had previously been used in earlier growth centile related research. As part of that process, we had applied the GROW customised calculator, which automatically flags and identifies implausible birthweights. This formed the basis of our exclusion criteria. We have now clarified this in the text (Methods, page 12).

*“Implausible birthweights had been previously identified within the dataset after application of GROW customised centiles for prior research, which automatically fails to generate a centile for implausible birthweights (34, 35).”*

8) **Page 21 "the introduction of this easily adoptable chart…" The authors make this repeated claim throughout the discussion, but they do not explain how this is easy to adopt.**

Thank you for drawing our attention to this. By ‘easy to adopt’, we mean that it could theoretically be adapted for use by any given population, by altering the mean birthweight and standard deviation in the algorithm. This could be done either using the calculator link embedded within the manuscript link, or by using the formula within the text. We have removed the phrase ‘easily adoptable chart’ and clarified how it could be translated to other populations if the appropriate mean and standard deviations are known (Discussion, page 24).

*“The introduction of this novel chart may enable clinicians to better identify fetuses at risk, offering surveillance and timely intervention to reduce this risk. The FROGS algorithm is able to be adapted to other local populations, if the population mean and standard deviation are known.”*

9) **Page 22 "it can also be updated easily if local mean birthweights change with time" The authors contradict their own earlier statement that this would not change with time. For an organisation to periodically check their population birthweight distributions there has to be academic infrastructure and support. Adapting local tools for reporting centiles is not that easy.**

Thank you for raising this. We agree with the reviewer in this comment, and earlier comments, that practice change is not ‘easy.’ As discussed above, we have removed references FROGS ‘not changing with time.’ We have also amended the sentence to *“It can also be updated if local mean birthweights change over time,”* which removes reference to an easy translation (Discussion, page 26). We have also now clarified the sentence in Discussion page 25 to *“While the FROGS algorithm is transparent and stable, the derived centiles can be updated if local mean birthweights change over time.”*

10) **Page 24 "FROGS can be freely and easily adapted…" The authors repeatedly state this apparent ease but they have not explained how this is easy.**

Please see response to Reviewer 1 point 4 and point 5.

**REVIEWER #2 COMMENTS:**

**Reviewer #2: Thank you for the opportunity to review this article by Pritchard et al. on a the Fetal Region-specific Optimised Growth Standard (FROGS), a fetal and birthweight centile calculator validated in a national population. The authors propose to adopt Hadlock's fetal weight formula and use a proportionality approach to "adjust" for the population-specific birthweight distribution. Mean term Australian birthweight was expressed as a proportion of Hadlock's mean birthweight. The calculated proportion/ratio is maintained at earlier gestations and standard deviations (expressed as a percentage of mean birthweight) were assumed to remain constant across GA. Adjustment for fetal sex is optionable. In the second part, the authors compared their chart to three widely adopted alternative charts in two ways: a "statistical validation", in which they compare the % of infants below several pre-defined thresholds, and an "outcome-based" validation in which the authors compare the ability of each chart to predict adverse outcomes associated with FGR, the primary outcome being 'stillbirth.**

**The article is an example of our continued struggle to define appropriate boundaries for normal fetal growth and - by extension - birthweight. Comparative studies are very difficult to interpret because of the many methodological differences between different charts. The main strength of this study is that it provides a simple tool which can easily adopted to other populations, provided that mean and SD of birthweight are available for this population. Another strength is that the charts were compared to two other charts with shared features (i.e., the incorporation of Hadlock's EFW formula), which minimizes the bias introduced by different methodologies, increasing the chance that observed differences are indeed attributable to differences in birthweight. The comparison to Hadlock illustrates the effect of adjustment for Australian mean birthweights and fetal sex; the comparison to the global fetal and birthweight calculator illustrates the effect of adjustment for fetal sex and the importance of classifying centiles according to each day. I have a few major comments which I think should be addressed before publication may be considered:**

1. **The primary outcome is stillbirth, for which both gestational and birthweight may be unreliable. How do the authors think this influenced their results? The association with perinatal death is also determined mostly by stillbirths, and much less by neonatal deaths.**

Thank you for raising this potential bias. We agree that gestational age at birth may not fully represent the gestational age at which fetal death occurred (which, in Australia, is on average several days prior). Additionally, weight for a stillborn infant may differ in utero compared to at the time of delivery, particularly with fluid loss or gains. This is a bias, and we have now discussed this in our limitations section (Discussion, page 27). However, as we applied all of our growth standards to every infant within our dataset, we would expect the same limitation to be present across all analyses, and thus to have a minimal impact on our overall findings. Additionally, we have many secondary outcomes that consistently demonstrate the same overall findings, and support the validity of our work.

*“Furthermore, when examining stillbirth, the gestational age and birthweight at birth may not accurately reflect the exact gestational age and birthweight at the time of fetal death, which may introduce bias. However, we would expect this bias to be equal across all growth standards applied to our dataset.”*

We also acknowledge that the perinatal death rate is heavily influenced by stillbirths. In Australia, the most recently published statistics suggested that 77% of all perinatal deaths were stillbirths (<https://www.aihw.gov.au/reports/mothers-babies/australias-mothers-babies/contents/stillbirths-neonatal-deaths/preliminary-perinatal-deaths>). Our dataset showed similar numbers, with 19% of the total perinatal deaths occurring post birth. We believe that the underrepresentation of neonatal deaths in the perinatal death rates seen in our SGA populations may be because neonatal deaths are more heavily influenced by prematurity than placental insufficiency, and so may not be as commonly seen in our studied SGA populations as stillbirth.

2. **The overall mortality rate was 0.25%, whereas the stillbirth rates in all SGA groups were higher: 0.85% for the AIWH, 1.02% by the Hadlock birthweight chart, 1.05% by the global chart and 1.1% by FROGS. What was the overall RR of SGA according to each chart, compared to non-SGA infants?**

Thank you for raising this point, and we are very happy to include this information. We have now added in the overall relative risk (RR) of SGA according to each chart, compared to non-SGA infants under Results, Outcome based validation of the FROGS chart (page 19).

RR SGA AIHW charts: 3.37 (95% CI 3.15 – 3.60)

RR SGA Hadlock charts: 6.51 (95% CI 5.95 – 7.12)

RR SGA Global charts: 6.69 (95% CI 6.11 – 7.32)

RR SGA FROGS charts: 7.05 (95% CI 6.45 – 7.72)

3. **Looking at the non-overlapping SGA populations, only those 'SGA by FROGS but not AIHW' have an absolute mortality rate that is higher than the "base" stillbirth rate in SGA infants; in the other two comparisons this risk was lower than the base rate in SGA infants. On the other hand, the 'extra' SGA infants who were 'overlooked' by FROGS but identified by the global standard had a stillbirth risk higher than the base stillbirth risk. Are 'extra' SGA infants at increased risk compared to the non-SGA population? I.e., is there a**
4. **n actual benefit for the new charts compared to already existing charts? And is this risk clinically relevant? Not just in terms of relative risk, but absolute differences, which may be different across gestational age? There is substantial evidence that the discriminative power of all charts, birthweight or fetal weight, are poor, with AUCs around 0.60. These nuances are important to show.**

Thank you for these comments, and apologies for not including the baseline stillbirth risk seen in the non-SGA population. It is true that each of the sub-populations identified by only one chart had a lower absolute stillbirth rate than that of the stillbirth rate seen in the total <10^th^ centile population. However, this is not unexpected. The very smallest, highest risk infants would be classified as small by any growth standard. Those infants classified by one chart but not another, will inherently be on the upper end of the <10^th^ centile range. Their risk of stillbirth is therefore likely to be much lower than infants born <3^rd^ centile, for example, which would be identified as SGA by any growth standard. However, we confirm that the stillbirth rate amongst the subgroups classified by one chart but not another are still higher than non-SGA infants, and so we believe this findings remains clinically relevant. We have now added the stillbirth rate for non-SGA infants in the Results page 19 to demonstrate that FROGS is still identifying an additional cohort of small infants at increased risk of stillbirth compared to non-SGA infants (Results, page 19).

*“Comparatively, the risk of stillbirth for an infant not classified as small for gestational age by any chart, was 1.5 per thousand.”*

We also agree that the assessment of fetal size in isolation has limitations in detecting fetal growth restriction. However, ultrasound derived EFW centile is a common entry pathway to increased surveillance and targeted management in all international guidelines. Hence, to optimise the yield of fetal size assessment it seems sensible to apply the growth chart with the best discriminative ability to identify infants at risk.

5. **The authors use a birthweight chart from AIHW and conclude that these "fail to identify the disproportionate number of preterm births that are small for gestational age". It is inherent to the design of a population-based birthweight chart to have low sensitivity for detecting infants with growth abnormalities, as such infants were all included. I wonder why the authors chose to use these birthweight charts, especially since there is an alternative Australian birthweight chart available (10.5694/mja2.50676) which aims to address this issue by excluding all births initiated by obstetric intervention, to minimize the influence of decisions to deliver SGA babies before term.**

Thank you for suggesting an alternative growth chart and highlighting the known issues with applying a birthweight chart to a preterm population. We are aware of the spontaneous birthweight centile charts published by Joseph et al. in 2020. They derived growth standards from Australian birthweight data covering the same time period as our study (2003-2013), but included only births in which labour was spontaneous, rather than induced or by caesarean section. While we believe the spontaneous birthweight centile charts are a valuable addition to the literature in proof of principle, we believe they have limited applicability as a comparator in our study:

There are three main reasons why we did not compare our proposed chart with this growth chart. First, in a preterm population, there are several studies suggesting that pregnancies complicated by placental insufficiency are still more likely to go into spontaneous preterm labour than their normally grown counterparts (Morgan et al. 2014 ‘Placental Insufficiency is a Leading Cause of Preterm Labour’). Second, population birthweight centile charts are currently used in Australia to report the weight of infants after birth – which means that management and delivery decisions will be made on an estimated fetal weight centile but then audited postnatally on a birthweight centile. We therefore chose to compare FROGS to the birthweight centile that most closely represented what is in current use for postnatal reporting.

Third, and most importantly, the published centile charts by Joseph et al. are hard to apply in a practical sense as they publish limited centiles only, based on the first day only of a completed week of gestation. For example, they publish the birthweight centile for 32+0 weeks gestation, 33+0 weeks gestation, etc. rather than by each gestational day or the average of all infants born at 32+0 to 32+6 weeks gestation, as is common in other birthweight charts. To apply this chart you would either have to a) apply the value at 32+0 to all infants born at 32+0 – 32+6 (which would underestimate the centile thresholds), or b) average the values for the first day of the week before and after to ‘approximate’ a midpoint. This may not be applying the centile charts in the way the original authors intended.

6. **The difference between the 10th percentile of this "prescriptive" chart and the AIHW chart is large, especially in preterm infants. The authors argue in their discussion that "prescriptive charts are not centered upon population birthweight means", but Hadlock's formula was also based on healthy women and in the Technical Appendix in the Supplemental material it is also stated that mean birthweight and SD should be derived from a population without risk factors for abnormal fetal growth. 5. The methodology is based on assumptions: e.g. that the shape of the Hadlock curve is appropriate, that the proportional effect of population characteristics is constant across gestational age, and that SD remains constant across GA. The assumptions were not tested and I think this requires attention in the discussion section. Simply stating that others have used a similar approach is not sufficient, and the fact that these results were published in a high-impact journal does not change this.**

Thank you for raising this point. We have changed the terminology in our manuscript to clarify that prescriptive charts are not necessarily centered upon population birthweight means (Discussion, page 25). We have also clarified and added that other research has demonstrated that many of the benefits of a prescriptive chart are observed simply when an intrauterine growth standard is used (Discussion, page 25).

*“Even if prescriptive charts are used that are representative of a given population, much of the benefit is simply seen with the use of a fetal, rather than birthweight chart (70). There is limited additional gain from restriction to a healthy population (70).”*

It is also a very valid point to raise that simply because the Hadlock curve and its derivative charts (eg. The global fetal and birthweight centile calculator and GROW customised charts) use the Hadlock shape, this does not confirm that is the most appropriate curve. We acknowledge that the gold standard growth chart for any population would likely be a large, prospectively collected set of growth percentiles derived from very frequent ultrasound measurements across gestation. However, the impracticality of this approach in many jurisdictions has meant that alternative, simpler options have been essential. We propose one such option. Prospective validation, by applying it to ultrasound estimated fetal weights and showing an appropriate distribution across gestation, would be required to validate the shape of the curve further. We have recommended this approach as an avenue for future research (Discussion, Strengths and Limitations, page 27).

*“A limitation of the FROGS growth standard is that it works under several assumptions; that the shape of the Hadlock curve is reasonable, that the birthweights are normally distributed, and that the standard deviation remains in a constant proportion to the mean across gestations. It is possible that these assumptions do not hold in all circumstances (73).” Prospective application of FROGs to an ultrasound population to test these assumptions is an important next step.”*

**Minor comments:**

7. **How do the authors explain the disproportionately large number of infants with a birthweight >p95 in a population born between 2009-2019, when mean and SD of birthweight are from 2019 data? Both mean and SD are influenced by the inclusion of large infants? This should be discussed in more detail.**

Thank you for raising this question. The mean and standard deviation are indeed influenced by the inclusion of very large infants. The fact that there is still a much greater proportion of infants born >95^th^ centile demonstrates that the data has a larger right tail and is therefore not completely normally distributed. We believe that this likely represents significant maternal demographic shifts during the decades since Hadlock’s original curve was created. This has been observed and reported in other studies (Park et al. 2018, Epidemic of large babies highlighted by use of INTERGROWTH-21^st^ international standard; Grantz et al. 2023 A new method for customized fetal growth reference percentiles).

Additionally, as reported in other studies (eg. Grantz et al. 2023 A new method for customized fetal growth reference percentiles), the extremes of observed birthweight can be more widely distributed than predicted distributions. This is because any growth standard that uses measures of central tendency will well-align with observed distributions at the centre of the data, but if there are particularly large left or right tails, as is the case in our data, then you can observe a greater proportion of infants than expected above these threshold extremes.

We carefully considered the implications of the large proportion of infants born with birthweight >95^th^ centile. We believe they are likely heavily influenced by non-physiological changes in maternal demographics, so have not altered the upper threshold limits, particularly given that clinical decision making around large infants is generally dictated by absolute size, rather than EFW centile. We have amended our Discussion to reflect this, page 28, as below.

*“FROGS classified more than expected >90^th^ and >97^th^ centile. FROGS used mean and standard deviation to identify the spread of percentiles, and this demonstrates that the upper extremes of birthweight are more widely distributed than predicted distribution (72). This overclassification could be seen as a limitation. However, this likely represents a genuine increase in the proportion of large infants in Australia (68), driven by increased maternal obesity (73-76) and diabetes (77, 78), as has been previously reported****.”***

8. **The Results section contains a lot of repetition of information that is also available in the tables/figures. Some results are already discussed or interpreted; this belongs in the Discussion section.**

Thank you. We have reviewed the results section and moved any interpretive comment to discussion.

9. **It would greatly help my understanding of the results if the different charts (i.e., the 10th percentiles) could be plotted in a graph, instead of (just) these Venn diagrams. This would help understand which infants are supposed to benefit from this new chart**

Thank you for raising this. We are very happy to include these additional diagrams, and have included graphs of the 10^th^ centile across gestation for both male and female infants. However, it is worth nothing that the differences between a week and day based chart will not be seen on these smoothed centiles. We have stratified by gestation so that any differences can be more easily seen and have included them as a supplementary figure (Supplmentary Figure 1A-F).

10. **There is an error in the section that compares Hadlock with the FROGS chart, where it says that 874,126 infants that were classified..**

Apologies for this error and thank you for identifying this. This has been corrected, now 73,379.

11. **Supplementary Table 1. should contain more information on the distribution of gestational age.**

Thank you for noting this. We have added further information to Supplementary Table 1 about the distribution of gestational age (stratifying into common gestational age brackets) (Tables, page 34).

12. **I am confused with regard to the 'confidence interval'. I was aware that 10% measurement error is generally accepted for estimations of fetal weight (which, for birthweight, would be unacceptable! Imagine an infant with a birthweight of 3500 grams, who weighs 3150 grams on one scale and 3850 gram on another!). "Assuming a 10% measurement  error, the true estimated fetal weight centile would fall between the Xth and Yth percentile". I tried the calculator for 2 term male infants: 3550 grams at 38+6 weeks and 3720 grams at 41+3 weeks. X and Y were p41.2 and p95 for the former, and p15.4 and p75.0 for the latter. What message do I take from this?**

We appreciate the reviewer's concern highlighting our use of the term 'confidence interval' and its associated statistical meaning. Our intention was for this term to represent the true uncertainty in the calculated birthweight centile due to underlying ultrasound measurement error. We understand however that the term 'confidence interval' may cause confusion by implying a specific probability distribution, which was not our intention. For this reason, we have renamed the term 'estimate range' in the revised manuscript to avoid confusion.

We also appreciate that at term, a 10% error can be quite significant and lead to significant variations in centiles. Your example highlights the common clinical conundrum where reported EFW centiles at term can vary widely with only 10% measurement error. It may be useful for clinicians to understand the limitations of size interpretation late in pregnancy. However, we believe this estimate range will be narrower at earlier gestations (when ultrasound derived fetal size is more likely to inform clinical surveillance and management decisions), and at the extremes of birthweight. For example, a 970g female infant at 28+4 weeks gestation would likely fall on the 2^nd^ centile, with an estimate range from <1^st^ to 10^th^ centile. This finding would imply that this is highly likely to be a small infant, even accounting for measurement error.

**REVIEWER # 3 COMMENTS:**

**Reviewer #3: This paper is concerned with developing a birthweight calculator. The proposed model seems like a simple yet reasonable adjustment of previously developed calculators and the development of a SAP in advance of data cleaning and analysis is desirable. Specific comments are given below.**

1. **Please discuss the statistical aspects of the estimation process since the description on page 12 is somewhat vague. Are the data spread a bit too thin when looking at centiles? Was there an implicit bias-variance (sometimes called calibration-sharpness) trade off when trying to adjust to all those targets? How was this best assessed?**

We appreciate the reviewer’s feedback on the statistical aspects of the FROGS standard and the opportunity to clarify how the model has been validated. As mentioned by the reviewer, the FROGS standard is based on an independent established formula, as initially proposed by Hadlock. The FROGS standard is a modification of this established Hadlock formula, which operates by adding a weighting based on the specified mean and standard deviation of the population birthweight distribution. A similar approach has been used previously, for example by Mikolajczyk et al. (A global reference for fetal-weight and birthweight percentiles – https://pubmed.ncbi.nlm.nih.gov/21621717/), with the aim of better aligning the growth standard with population birthweight distributions.

As such, the FROGS standard is not adjusted for any specific centile targets and instead relies on incorporating the mean and standard deviation of the intended population’s birthweight to alter the centile distribution. Particularly, in our work we used fixed values for the mean and standard deviation of the population birthweight which were taken from a separate data source, the Australian Institute of Health and Welfare (AIHW) as described on page 9 of the updated manuscript.

As such, no adjustment was performed with respect to the FROGS growth standard to align with a specific target. The intention of the work was instead to measure the performance of the various growth standards, including the proposed FROGS standards, against a set of fixed centile thresholds with clinical relevance. We took a similar approach to the FIGO recommendation for validation – “Identifying the chart that when applied to the local population yields weight percentiles that follow a normal distribution centered at approximately the 50^th^ percentile, and identifies approximately 10% of the low-risk population as being below the 10^th^ percentile and above the 90th percentile, and approximately 5% of the population as being below the 5^th^ percentile and above the 95^th^ percentile” (<https://pubmed.ncbi.nlm.nih.gov/33740264/>).

To ensure this is clear in the updated manuscript, we have clarified this further in Methods on page 13 and 14, as below.

*“The aim of the statistical validation of the charts was to a) Compare the overall distribution of the population birthweight centiles, as classified by each growth standard and b) Assess the proportion of infants born <3^rd^ centile, <10^th^ centile, <50^th^, >90^th^ centile and >97^th^ centile by FROGS and all other charts These centile cutoffs were used because they are clinically relevant thresholds which impact management (1, 2). We also assessed the distribution of birthweight centiles visually using a histogram and compared the mean, median and 25^th^-75^th^ centile ranges. This approach was in line with the approach recommended by FIGO(18).”*

2. **The performance of the FROGS model in the >95% percentile appears unexpectedly poor, please discuss the potential reasons for this (beyond the short discussion at the end of page 23) and how the model may be amended to correct this behaviour.**

Thank you for raising this excellent point, which was also raised by Reviewer 2, point 6. It has been addressed completely in our response on page 10 of this letter.

3. **Please add a critical discussion of 1) the selection of the comparators, especially beyond the global chart compared to their current usage worldwide and 2) the training and test sets and how they relate to the comparators' performance. For example, the authors use Australian mean birthweight and standard deviation for the multiplicative adjustment and then test their model to Australian data, is this correct? Typically, independent or external validation (i.e. against a country whose data have not been used) would be seen as a robust test of the model's performance. At the very least, the presentation and discussion should reflect this practice as appropriate, especially since PLoS Medicine takes a global view to public health so the utility of FROGS outside Australia should be discussed fairly.**

Thank you for these valuable questions and comments. Regarding the first point on comparator charts, these were largely chosen to help us elicit whether a certain attribute of FROGS was beneficial. For example, comparison to the original Hadlock chart demonstrates the impact of adjusting for population means and fetal sex, comparison to the AIHW charts demonstrates the benefit of using a fetal growth standard, and comparison to global charts highlights the differences seen when adjusting for gestational age in days and fetal sex. We have now added an additional paragraph in our discussion to further highlight the rationale behind our comparator charts (Discussion, page 28).

*“One of the strengths of our study is the comparison of the FROGS algorithm to other charts with shared features. This helped us to draw conclusions about a specific attribute of FROGS that may have been beneficial. For example, comparison to the original Hadlock formula highlighted the differences if population birthweight and fetal sex are adjusted for, while comparison to a population-based birthweight chart reinforced the benefits of using a fetal-weight standard. While use of these comparison charts has helped us clarify these benefits, the global utility of FROGS could be further investigated by comparing to local or international growth standards derived using different methodology.“*

With response to the second point, we confirm that the Australia mean and standard deviation birthweight was used as taken from the separate Australian Institute of Health and Welfare data source, and the FROGS standard was validated on an Australian dataset. This in an intentional feature of the FROGS standard to use the population mean and standard deviation birthweight. For example, if the FROGS algorithm was to be tested in an external population such as another country (eg. New Zealand), we would expect that the FROGS algorithm would then be adjusted for New Zealand mean birthweight, so that it is representative of that population.

We agree with the reviewer however that this should be explicitly highlighted and have added to the discussion that the FROGS standard should be further validated in external international populations, as adjusting for their local birthweights and standard deviations (Discussion, Research Implications, page 28).

*“While it may be common for a statistical model to be developed in one population and validated in an external population, the design of FROGS is such that adjusting for a local population mean and standard deviation is necessary. Further research should include validating the FROGS in external populations, using their local population birthweight mean and standard deviation to confirm international applicability.”*

4. **When focusing on the prediction of a particular target, say the population that is characterised as small for gestational age, there are two types of error that may occur, pertaining to sensitivity and specificity (or false positives/negatives). Essentially, one may have an interval that is too wide and always contain the small children, or having an interval that contains exclusively small children but may miss some. Is this a concern in this prediction task and if so should the reporting focus on both aspects?**

Thank you for raising this point, which is of particular importance in discussing the use of size as a surrogate marker for fetal growth restriction. One of the strengths of our study is that the proportions of infants classified as <10^th^ centile according to all standards were quite similar in absolute size (10.4% FROGS, 9.3% AIHW, 11.1% Hadlock, 10.9% global). This meant that all charts had a similar screen positive rate. Presenting the relative risk (RR) within these similarly sized screen positive groups provides a measure of the chart’s performance which aligns with its sensitivity/specificity. i.e. increased sensitivity and specificity will result in increased relative risk.

To clarify this point, we have added the overall relative risk of stillbirth if classified as <10^th^ centile by each chart (see response to reviewer 2 point 2, page 7) which we believe, in the context of similar screen positive rates, helps highlight the clinical value of each given chart. We have added this to the manuscript Discussion on page 19.

“The relative risk of stillbirth if an infant was SGA compared with non-SGA was 3.37 (95% CI 3.15 – 3.60) for AIHW charts, 6.51 (95% CI 5.95 – 7.12) for Hadlock charts, 6.69 (95% CI 6.11 – 7.32) for global charts, and 7.05 (95% CI 6.45 – 7.72) for FROGS.”

5. **Please follow standard scientific practice and use precise/rigorous language without overstating the study findings. For example, a confidence interval refers to the frequentist quantification of parameter (and not model) uncertainty under repeated sampling. Accounting for arbitrary measurement error of a single factor does not yield confidence intervals. In some scientific areas this would be called  perturbation analysis or one-way deterministic sensitivity analysis, but this "interval" may not be associated with a nominal coverage of any sort.**

We appreciate and agree with the reviewer's concern highlighting our use of the term 'confidence interval' and its associated statistical meaning. Our intention was for this term to represent the uncertainty in the calculated birthweight centile due to underlying ultrasound measurement error, as outlined in our response to Reviewer 2 point 11. We appreciate the use of the term ‘confidence interval’ was unhelpful and confusing in this context and have updated this term to 'estimate range' in the revised manuscript (such as in Methods, Part 1, 9).

6. **Similarly, exact centiles suggest lack of approximation of any sort, a practically impossible task in any estimate, especially by the deterministic model proposed in this paper. I am a statistician and not a gynecologist but I understand that the exact conception day is typically unknown. If this is true, then the claim for accuracy by day within the gestational week is less relevant perhaps?**

Thank you for raising this, and it is absolutely true that the exact conception day can never be precisely known. The estimated due date in our study population is derived from a combination of menstrual dating, and early pregnancy ultrasounds, which is commonly performed in Australia. Therefore, we would expect reasonable accuracy in determining the estimated due date. Regardless, even if the accuracy of the estimated due date is in question, we believe there is still benefit to a more precise measure of gestational days rather than weeks. This is because, within a pregnancy, there are often multiple ultrasound measurements taken over time. It is important that those measurements are precise in relation to each other, in order to accurately assess the growth trajectory over time. We have demonstrated previously that there can be very significant variations if gestational age in weeks only is used (https://journals.plos.org/plosone/article?id=10.1371/journal.pone.0271538).

**REVIEWER #4 COMMENTS:**

**Reviewer #4: This paper addresses an important and controversial topic. I think the goal is to have a published paper to adopt this process in Australia. I think the paper needs to have slightly more nuanced conclusions and clarify the methods as outlined above.**

Thank you for these comments. We agree that it would be our hope to have this growth standard adopted within Australia. However, we have specifically aimed for publication in an international journal because we believe our formula offers one of the least complex growth standards to adapt to external populations, by using relatively easy to obtain population statistics. FROGS may therefore have implications for international research collaborations, or for clinical use in countries that do not have external validated growth standards in use. We have highlighted potential international implications at various points in our discussion, such as the below examples from Discussion page 28 and 29.

*“Our obstetric growth standard can be freely translated anywhere, by adjusting the mean birthweight and standard deviation. Further research should include validating the FROGS in external populations, using their local population birthweight mean and standard deviation to confirm international applicability.”*

*“While use of these comparison charts has helped us clarify these benefits, the global utility of FROGS could be further investigated by comparing to local or international growth standards derived using different methodology.”*

**Introduction**

1. **The 2nd half of this sentence that states: "International [sic] guidelines also recommend that an obstetric growth curve represents the local population it is used in. (Ref 18, 27)" is not entirely technically precise. FIGO (Ref 18) concludes that "Alternatively, international standards for growth may be used with locally appropriate thresholds for risk interpretation" meaning that the Hadlock 1991 reference could be used if the cutoffs selected to define SGA or LGA are tailored to a local population. I do think this summary of FIGO recommendations in the Introduction should be edited to be technically precise.**

Thank you for this clarification; we did not intend to misrepresent the FIGO guidelines. We have altered our introduction to read *“international guidelines also recommend that an obstetric growth curve represents the local population it is used in if traditional centile thresholds (such as <10^th^ centile) are to be used (18, 27).”*

2. **The statement that "This instability makes these growth standards very difficult to apply in databases, research settings, or to use at a population level." Is somewhat true, but I would argue that they are not "very difficult". Grantz et al has published a heteroscedastic model to easily implement customized growth charts (A new method for customized fetal growth reference percentiles. PLoS One. 2023;18(3):e0282791. PMID: 36928064.) and Jason Gardosi's customization can be purchased for clinical use - albeit cost might be an issue. Also, "have algorithms hidden by the institutes that derive them" is somewhat true although the customization method has been published multiple times by Gardosi et al and can be easily implemented (as per the Grantz paper). In fact, many countries use customized fetal growth charts (McCowan ref). The main disadvantage of customization is that it that there is controversy as to whether it improves detection of fetuses at risk for perinatal morbidity and mortality (Zhang, Carberry) Suggest editing these sentences to be more technically precise.**

Thank you for highlighting some of the issues with the terminology used in our manuscript. We agree that the GROW software can be purchased for clinical use, and so the term ‘very difficult’ is not accurate. However, as a research team that has worked with the GROW software over the past decade, we have ourselves noticed difficulties when applying it – the bulk centile calculator (used for research) is frequently updated, with previous iterations decommissioned. The centiles that are derived from each new version differ, with no real way of accessing the previous version or knowing why the changes have occurred. We have changed the introduction text to more precisely reflect our concerns, as below (Introduction, page 6).

*“Customised growth standards are also complex, change over time, have algorithms hidden by the institutes that derive them, and have a cost associated with their use that may preclude access in many settings (30). The fact that the customised centile calculator varies across years, without the contributors to the variance always being clearly described, can make them difficult to use in research settings, or when auditing population changes over time.”*

We have also discussed the controversy in our discussion, referencing the commentary by Zhang et al. and research by Carberry et al. (Discussion, page 24). However, we have tried to keep it brief as we are aware that there is already extensive published literature on the advantages and disadvantages of customisation.

*“Whether or not to customise centiles for maternal characteristics is potentially the most contentious issue (19, 26). Full customisation based on maternal characteristics including maternal height, weight, parity and ethnicity may help identify infants at higher risk of adverse perinatal outcomes (53-55). However there has been significant controversy as to whether the potentially marginal gains of full customisation are worth the complexity of the algorithms, above an uncustomised population-based chart (56, 57). Customisation has many additional limitations. The algorithms used to derive customised growth standards are highly complex, change over time (where new chart updates are produced frequently), and the methods used to undertake the customisation are not always disclosed by the institutes that generate them (30). These factors can make customised growth standards difficult to apply clinically at a population level, or in research settings, as it can be hard to compare cohorts from different time points. Additionally, maternal height, weight and ethnicity are not purely physiological mediators of birthweight (58-63). This has incited debate and has hindered widespread adoption of customised charts (19, 30, 31, 56, 64). Furthermore, in a highly diverse society like Australia, adjusting for ethnicity is not always feasible (65).”*

**Methods**

3. **The proportionality assumption was recently examined and questioned by Grantz et al (A new method for customized fetal growth reference percentiles. PLoS One. 2023;18(3):e0282791. PMID: 36928064.) This potential limitation should be mentioned in the discussion.**

Thank you for noting this valuable reference. Grantz et al. examined the customised growth centile method proposed by Gardosi (proportionality method), as well as two further models, one that customised both mean and standard deviation while retaining the assumption of normality, and one that calculated customised percentiles and standard deviations using quantile regression without assuming normality. We would like to highlight a few points regarding this paper:

a) The main finding of this paper was that, if customisation is used, it is not only the mean but also the standard deviation that should be adjusted for when considering maternal characteristics. Ie. If you customise on a maternal characteristic, you should adjust both the weight and standard deviation for that characteristic. While this is very valuable information, it is less relevant for our study, in which we do not customise on maternal characteristics.
b) Fetal sex was not found in their paper to have any significant non-constant influence on the distribution of estimated fetal weight. This suggests that our approach may remain appropriate in the context of the variable fetal sex.
c) When comparing models that assumed a normal distribution of birthweight to a quantile regression model, the authors noted “the effect of non-normality did not have an appreciable impact on model performance.” This suggests that remaining with a simpler model may still be appropriate.

However, we agree with the reviewer that the proportionality assumption, and assumption of normality, have not been formally tested in our study. We have added this as a potential limitation in our discussion and have referenced the above study (Discussion, Strengths and Limitations, page 27).

*“A limitation of the FROGS growth standard is that it works under several assumptions; that the shape of the Hadlock curve is reasonable, the birthweights are normally distributed, and that the standard deviation remains in a constant proportion to the mean at all earlier gestations. It is possible that these assumptions do not hold in all circumstances (73).”*

4. **For part 1, the reference for the Hadlock EFW formula is incorrectly labeled. The formula presented is not to calculate EFW which would be a formula as published in the 1985 paper. The Hadlock 1991 paper, with the formula presented calculates "median fetal weight for each gestational week".**

Apologies for this error, and thank you for identifying this. We have changed the terminology and have the removed the words ‘estimated fetal weight’ so as to avoid confusion with the formula used to calculate EFW (page 7, Part 1 of methods).

5. **Also, the authors state "mean" throughout Part 1 but do they mean median or 50th percentile?**

Thank you for seeking clarification on this point. Mean is correct. This will also approximate the median in a normally distributed set of birthweights.

6. **For the Gardosi method, the standard deviation is not assumed to be "constant" as stated; rather, it's the coefficient of variation that is assumed to be constant. This should be corrected.**

Apologies for this error, and thank you for highlighting this issue. We have corrected the manuscript to clarify that it is the coefficient of variation, and the standard deviation expressed as a percentage of birthweight, that remains constant, rather than the standard deviation itself (Methods, page 9).

*“This percentage was used in all cases, as it was representative of both male (standard deviation 429g, 11.8%) and female (standard deviation 412g, 11.8%) infants. For all earlier gestations, the standard deviation used to derive percentiles remained constant at 11.8% of the mean birthweight for any given gestational age.”*

7. **It's not clear where the 3705 g for Hadlock was published. Per the Hadlock 1991 paper, the 50th percentile at 40 weeks was 3,619 g. Need to provide a reference.**

Thank you for identifying this. The 50^th^ percentile at 40 weeks was 3,619g. However, as per the Hadlock formula, this value is reached at 40+0 weeks gestation. The 3,705g is reached at the midpoint between 40 and 41 weeks’ gestation. We have now added a reference for this (Methods, page 8).

8. **I'm not sure this is a correct statement: "As identified in Hadlock's study, the standard deviation at term is applicable at preterm gestations." As I point out above, it's the coefficient of variation that is constant. The value of the SD depends on the mean EFW, so will most certainly vary depending on gestational age since fetuses are smaller on average preterm than at term.**

Thank you for raising this point, which is correct. The coefficient of variation is what is applicable at preterm gestations. We have clarified this in the manuscript (Methods, page 9).

*“For all earlier gestations, the standard deviation used to derive percentiles remained constant at 11.8% of the mean birthweight for any given gestational age.”*

9. **Along these lines, it's not clear what was done with the SD of 421 g - do the authors mean they used this SD at every gestational age? If so, that would not be correct. Or perhaps they mean they used the same SD regardless of fetal sex. The methods need to be clarified.**

Thank you for raising this point. We agree with the reviewer that the terminology was ambiguous. We did not mean that the absolute standard deviation of 421g was constant in all cases, but rather the standard deviation expressed as a percentage of birthweight (11.8%). As mentioned on page 9, that same percentage was relevant for both male and female infants (despite having standard deviations that were different in their absolute values). We have corrected this in the text (Paragraph 2, page 9), as highlighted in our response to Reviewer 1 point 6.

*“The standard deviation at 40 weeks’ gestation for both sexes combined was 421g, which, as a percentage of birthweight, was 11.8%. This percentage was used in all cases, as it was representative of both male (standard deviation 429g, 11.8%) and female (standard deviation 412g, 11.8%) infants.”*

10. **I defer to a statistician as to whether the calculation of the confidence interval incorporating 10% measurement error is correct. I'm not familiar with this procedure. In the very least, they probably should not refer to it as a confidence interval.**

We appreciate the reviewer's concern highlighting our use of the term 'confidence interval' and we have now amended the term to ‘estimate range’, as outlined in response to Reviewer 2 point 11.

11. **Since the authors are following FIGO guidelines for evaluating FROG, I don't understand the rationale behind comparing to birthweight charts since these are inherently flawed and aren't recommended for clinical use when monitoring fetal weight. The rationale given has already been thoroughly established. I'm not opposed to this exercise, but in multiple places in the paper including the abstract it's concluded that FROG is better than "x, y, z charts" - I don't think it's informative to include birthweight charts in the final conclusions.**

Thank you for raising this issue. We wholeheartedly agree that birthweight charts are flawed and unsuitable for use when measuring estimated fetal weight during pregnancy. However, it is currently mandatory to report the birthweight using Australian population birthweight charts, even at preterm gestations, which are subsequently used for research and policy purposes. Management decisions may have been initiated using a fetal chart, but then audited using a birthweight chart. This can lead to confusion and the artificial appearance that abnormal size was perhaps unidentified. While we are not necessarily advocating that FROGS be used for postnatal reporting, we do believe it helps highlight the differences between a fetal and birthweight chart, even if derived from the same mean and standard deviation. We have now clarified the discussion to acknowledge that the benefits of a fetal chart are already well established (Discussion, page 23).

*“First, charts based on in utero ultrasound measurements, rather than birthweights, better identify preterm infants who experienced pathological growth (25, 26). This is consistent with international research and guidelines (18, 27), and was evident when we compared the FROGS to the AIHW birthweight charts, which failed to identify many high-risk, preterm infants.”*

12. **The classification of fetal weight centiles using the "completed weeks" method is going to cause problems inherent to this method. The authors know that and conclude accordingly. However, in clinical practice, my understanding is that ultrasound software in clinical systems interpolate between the weeks so that a day specific EFW percentile is technically reported. So, it's an important issue for researchers to be aware of, but less so for clinical practice. The authors might at least expand upon this distinction in the discussion.**

Thank you for raising this point. While we know that some ultrasound software does automatically report exact centiles, there are also situations in which the derived estimated fetal weight is then applied to a different centile chart. We hope that by highlighting the clinical benefits of using exact days, it will help standardise best practice.

13. **It would be important to know how accurate and valid the data are by adding a sentence and a reference.**

Apologies for not including a statement on data accuracy. We have highlighted references to the accuracy of the data, which has previously been validated and tested in prior research (Discussion, page 11).

*Data quality has previously been audited and validated in prior research (43, 44).*

**Results**

14. **Table 2- it should be added to the footnote that these are based on birthweight (as opposed to fetal weight.)**

Apologies for this omission, we have added this footnote (Table 2).

15. **Table 2 - It's important to know which fetal growth chart tends to be used in clinical practice. It seems that part of the problem with comparing stillbirth between these 2 groups is that presumably the fetuses that were SGA by the AIHW and Hadlock charts (which are more like the un-customized fetal charts?) were more likely to have been identified antenatally and therefore monitored more closely and/or delivered to prevent a stillbirth. If customized charts aren't used antenatally, then these fetuses that were SGA by FROG but not AIHW may have been more likely to remain unidentified. Can the authors comment on this potential bias in the Discussion?**

Thank you for highlighting this issue. This is an important issue and bias, and we agree that if a certain chart was used in clinical practice then it is more likely to lead to identified, and managed, fetal growth restriction in which you would expect fewer adverse outcomes. Unfortunately, in Victoria, there are a multitude of different growth charts in clinical practice, which was part of the impetus behind our research. Charts that were in use over the study time period included Hadlock, a variation of the global fetal and birthweight calculator, and several variations of customised centiles. Postnatal reporting and audits are also usually presented using Australian birthweight charts. We have now added a section in the discussion to highlight this potential bias (Discussion, page 27). As there is currently no standard chart, we are unable to comment as to which direction the bias would likely lie.

*“Multiple growth standards were in clinical use in Victoria during the study timeframe. Fetuses that were identified as small on the chart in use would have been more likely to be identified antenatally and have risk mitigation strategies in place to reduce the risk of adverse outcomes. This may have affected our findings.”*

16. **These findings are supported by the fact that the FROGS and Global charts are more similar. (Although see my earlier comment about exact day versus completed weeks.)**

Thank you for these comments. We agree that day-based charts are of greater clinical value. For some time, a variation of the Global charts (a week-based chart) were used in Victoria in several institutions in clinical practice. Therefore we do believe there is some benefit in highlighting differences between day and week based charts.

**Discussion**

17. **The clinical implications are overstated given the limitations outlined above. The authors do call for prospective evaluation; however, it should be noted that an RCT would be needed to compare the charts, which has already been conducted and did not find an improvement in customization. (Matias Vieira et al. The DESiGN cluster randomised trial)**

We appreciate the reviewers comments on the overstatement of the clinical implications of our chart. We have amended our comments regarding the clinical implications at several points throughout the text. We have removed several statements suggesting that FROGS is easy to translate or adopt (Abstract page 3, Discussion page 21 and page 22) as we acknowledge that any potential clinical change can have significant challenges with implementation. We have changed the sentence *“FROGS offers improved research and clinical applications”* to *“FROGS offers potential research and clinical applications.”* In discussion page 28. We have added a further comment in the Discussion (page 25) to acknowledge pragmatic challenges to implementation, but explain why FROGS may still have some potential applications (Discussion, page 26).

*“While implementation of any clinical change always has pragmatic challenges, we believe that the ability of FROGS to generate a growth centile chart based on population-based birthweight data, one of the most widely accessible epidemiological data points, will limit the barriers to change.”*

Thank you also for highlighting the DESiGN cluster randomised trial, a pragmatic open cluster RCT with the intervention of a “Growth Assessment Protocol (GAP),” which included a series of interventions (including the use of customised centile charts) designed to improve detection of small for gestational age infants and thereby reduce stillbirth risk. We do note several design features about this study. First, both the customised and uncustomised growth standards compared in the trial were already based on UK population averages. One of the key findings of our study is that we agree that adjusting growth standards for population means will better improve clinical utility. Second, the growth chart was only one part of the set of interventions offered in the GAP protocol, and so the impact in isolation is hard to assess. Finally, the primary outcome was detection of SGA antenatally (SGA defined as small by both customised and uncustomised growth charts). Therefore we may expect that both charts perform equally well at detecting these unequivocally small infants.

We believe the DESiGN trial is of great value to the literature, but hope that the design of our study and the type of growth standards examined in our research may differ enough from the DESiGN trial to still warrant prospective evaluation in future.

**Minor**

18. **Suggest avoiding using "this" as a subject of a sentence because it's often not clear to the reader what "this" is referring to. Would replace "this" with a specific noun or pronoun that clearly identifies what the authors are referring to, depending on the context of your sentence. Examples: "This is a barrier…"; "To overcome this…"; "As this is a healthier cohort"; "However, this is not…"  in the Introduction, page 4-5 (multiple places) and in multiple other places throughout.**

Thank you for highlighting this confusing terminology. We have replaced ‘this’ with specific nouns or pronouns in all instances raised by the reviewer, and those identified within the introduction.

**GENERAL EDITORIAL COMMENTS:**

- We have amended the formatting of the abstract
- We have added a lay Author Summary below the abstract

***Why Was This Study Done?***

- *Growth centile curves are used in obstetrics, to identify small (particularly <10^th^ centile) or large fetuses at risk of complications.*
- *However, the optimal growth standard is still heavily debated, with many varied growth centile curves in current use.*
- *We designed a new growth standard (the Fetal Region-Specific Optimised Growth Standard (FROGS)) that incorporated features identified from prior research that best identify small infants at risk of complications. We validated it by comparing against alternative growth standards in existing use.*

***What Did the Researchers Do and Find?***

- *We designed and published the FROGS algorithm, which is an ultrasound-based growth curve, that can be adjusted for the mean birthweight and standard deviation of any given population, provides an exact centile for each gestational day, and is optionally adjustable for fetal sex.*
- *We compared FROGS to three other commonly used growth standards (Hadlock, a global fetal and birthweight centile, and Australian population charts). We found that all growth charts classified similar numbers of infants as <10^th^ centile.*
- *When comparing non-overlapping populations (infants considered small by one chart but not another), FROGS outperformed the other charts in identifying infants at risk of stillbirth and other adverse outcomes including small for gestational age, NICU admissions, low Apgar scores and operative delivery (caesarean section or instrumental birth) for suspected fetal compromise.*

***What Do These Findings Mean?***

- *Compared to currently used charts, the FROGS chart classifies a similar proportion of babies as small, but may better identify small infants at increased risk of stillbirth or other serious complications.*
- *The FROGS algorithm can be adapted to other local populations, or applied to global research settings.*
